# Distribution of White-to-White Corneal Diameter and Anterior Chamber Depth in Chinese Myopic Patients

**DOI:** 10.3389/fmed.2021.732719

**Published:** 2021-11-18

**Authors:** Guihua Xu, Guanrong Wu, Zijing Du, Shanqing Zhu, Yunxiang Guo, Honghua Yu, Yijun Hu

**Affiliations:** ^1^Department of Ophthalmology, Huizhou Municipal Central Hospital, Huizhou, China; ^2^Department of Ophthalmology, Guangdong Eye Institute, Guangdong Provincial People's Hospital, Guangdong Academy of Medical Sciences, Guangzhou, China; ^3^Aier Institute of Refractive Surgery, Refractive Surgery Center, Guangzhou Aier Eye Hospital, Guangzhou, China; ^4^Aier School of Ophthalmology, Central South University, Changsha, China

**Keywords:** distribution, white-to-white corneal diameter, anterior chamber depth, Chinese, myopia patients

## Abstract

**Purpose:** To investigate the distribution of white-to-white (WTW) corneal diameter and anterior chamber depth (ACD) in Chinese myopia patients.

**Methods:** This was a cross-sectional observational study conducted at five ophthalmic centers. Anterior segment biometry was performed in 7,893 eyes of the 7,893 myopic patients using Pentacam, and the WTW and ACD were recorded. The distribution patterns of WTW and ACD were evaluated and the correlation between WTW and ACD was analyzed statistically.

**Results:** There were 4416 (55.95%) males and 3477 (44.05%) females. The age of the study population was 25.14 ± 5.41 years. Distribution of WTW was slightly positively skewed (Skewness = 0.0076, Kurtosis = 0.3944, KS *P* = 0.020) with a mean of 11.65 ± 0.38 mm and a 95% normal range of 10.91–12.39 mm. A significant difference in WTW was found among different myopia groups (*P* < 0.001). The ACD was normally distributed (Skewness = 0.899, Kurtosis = 0.027, KS *P* = 0.086). The mean ACD was 3.25 ± 0.26 mm and the 95% normal range of was 2.74–3.75 mm. A significant difference in ACD was also found among different myopia groups (*P* = 0.030). There was a significant correlation between WTW and ACD (*r* = 0.460, *P* < 0.001).

**Conclusions:** In our study, 95% of the Chinese myopic patients had a WTW within 10.91–12.39 mm and an ACD within 2.74–3.75 mm. ACD and WTW were significantly different among different myopia, gender and age groups. WTW was positively correlated with ACD.

## Introduction

Anterior segment parameters such as white-to-white (WTW) corneal diameter and anterior chamber depth (ACD) are not only of essential importance for preoperative evaluation of refractive surgery, but also provide crucial information about individual ocular anatomy (1–3). Accurate measurement of WTW and ACD is needed for implantable collamer lens (ICL) sizing before surgery (4). Implantation of an incorrect size ICL may lead to complications after the surgery, such as corneal endothelial damage, uveitis, and glaucoma (4). Besides, shallow central ACD was found to be an independent predictive risk factor for the development of any form of angle closure in the Handan Eye study (5) and in the Namil Study (6). Thus, it is important to identify such patients so that prophylactical treatment can be used to prevent an acute attack of angle-closure and primary angle-closure glaucoma (PACG). As for the WTW corneal diameter, an abnormal WTW may indicate corneal diseases like micro-cornea, microphthalmos, and so on (7). It is important to recognize these conditions before ocular surgery as they may be associated with other ocular and systemic disorders that compromise the outcomes of the surgery. WTW and ACD are also important parameters in some formulas used to calculate the power of intraocular lens (IOL) for cataract surgery (8, 9).

Previous studies have been carried out to investigate anterior segment parameters in various populations (10–13). However, results from these reports may not be directly applied to our patients due to possible ethnical differences in the anterior segment anatomy (1). In a recent study, the distribution of WTW was investigated in Chinese cataractous patients aged 63.7 ± 12.4 years (14). To date, little information is known about WTW and ACD distribution in young Chinese myopic adults, who represent the largest population of refractive surgery candidates in the world. In the present study, using pooled data from five ophthalmic centers we revealed the distribution patterns of WTW and ACD in this specific group of patients.

## Materials and Methods

### Participants

A total of 7,893 eyes of 7,893 myopic patients from Guangzhou Aier Eye Hospital (GZ), Shenyang Aier Eye Hospital (SY), Chengdu Aier Eye Hospital (CD), Wuhan Aier Eye Hospital (WH), and Hankou Aier Eye Hospital (HK) were retrospectively recruited. The study was approved by the Institutional Review Board (IRB) of every hospital (GZ, SY, CD, WH, and HK) and conducted in agreement with the Declaration of Helsinki. The IRBs waived the need of informed consent as the study only involved review and analysis of medical records and no individual patient could be identified from the data (15, 16). Inclusion criteria were myopic patients with a spherical equivalent (SE) ≤ −0.50 D and good quality Scheimpflug scans. Only the right eyes of the patients were included for analysis. Exclusion criteria were coexisting corneal diseases, keratoconus, forme fruste keratoconus, severe dry eye, non-axial myopia (such as those caused by spherophakia), previous ocular trauma or surgery, uveitis, glaucoma, wearing contact lenses within the previous 2 weeks, age younger than 18 years (unstable refraction) or older than 40 years (to reduce the effects of the crystal lens on anterior chamber depth measurement) (15, 16).

### Examinations

All of the eyes underwent routine ophthalmic examinations including decimal visual acuity, intraocular pressure (IOP), cycloplegic and manifest refraction, anterior segment examination by slit-lamp, corneal topography and tomography (Pentacam). Clinical data of the eyes were retrieved from an electronic medical record database. The spherical equivalent (SE) was defined as “spherical error + 1/2 cylindrical error” using manifest refraction. The eyes were divided into four groups according to the manifest SE: low myopia (−3.00 D < SE ≤ −0.5 D, LM), moderate myopia (−6.00 D < SE ≤ −3.00 D, MM), high myopia (−10.00D < SE ≤ −6.00 D, HM) and extremely high myopia (SE ≤ −10 D, EHM).

**Figure 1:**
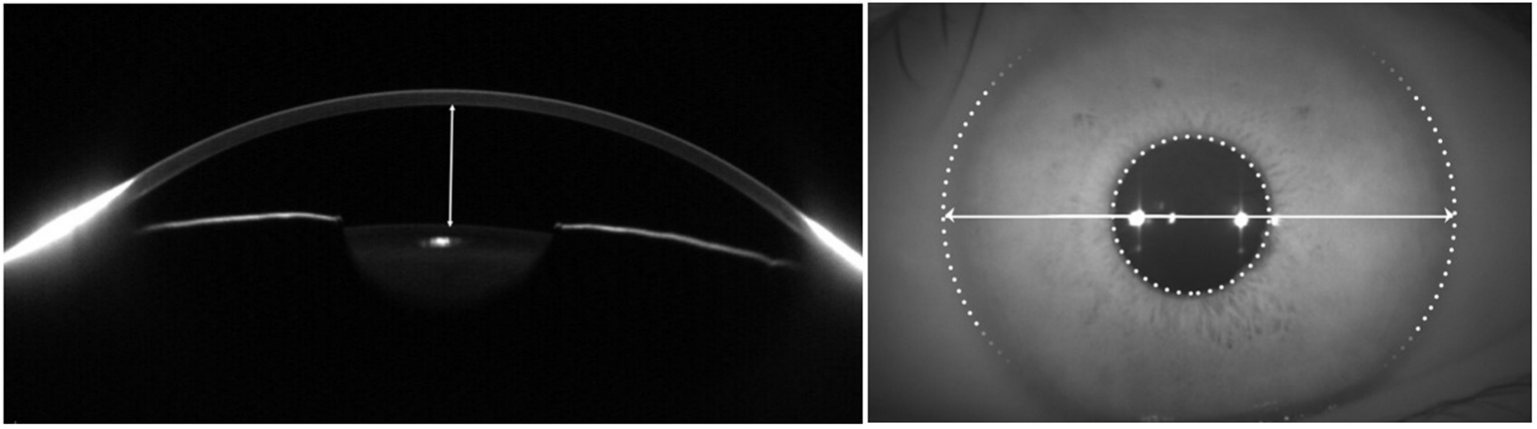
Delineations of white-to-white (WTW) corneal diameter and anterior chamber depth (ACD) measurements with Pentacam. The distance between the anterior surface of the crystalline lens and posterior surface of cornea was ACD (left) and WTW was the distance between nasal and temporal limbus points between the white sclera (right).

The WTW and ACD were measured with Pentacam by experienced technicians under dim light condition according to the standard procedures of the manufacturer (Oculus GmbH, Wetzlar, Germany) as previously described (15, 16). The fixation target was set to a viewing distance of +1.00 to 0.00 D (far vision) (17). Depth of anterior chamber (ACD) was the distance between the anterior surface of the crystalline lens and posterior surface of cornea, and WTW was the distance between nasal and temporal limbus points between the white sclera and the darker iris image (Figure 1). Repeated measurement was taken when Pentacam scans did not pass the quality check. Quality control and data retrieving of Pentacam examination were previously described (15, 16).

### Statistical Analysis

Pooled data of the five ophthalmic centers were used for analysis. Distribution of the WTW and ACD was evaluated by Kolmogorov–Smirnov (KS) test. Data of WTW, ACD, age, and SE were presented as mean ± standard deviation (SD). Kruskal–Wallis test was used for comparison of WTW and ACD among different myopia groups and age groups. Two sample *t*-test was used for ACD and WTW comparison between different genders among different myopia groups. Correlation between the WTW and ACD, between WTW/ACD and age, and between WTW/ACD and refractive error was evaluated by Spearman correlation test. *P* < 0.05 was considered to be statistically significant.

## Results

### Demography

There were 7,893 patients (7,893 eyes) included in the study and 55.95% of them were male. The mean age of the patients was 25.14 ± 5.41 years. The mean SE of the eyes was −4.87 ± 1.66 D. There was a significant difference in age, gender, SE, ACD, and WTW among patients from different ophthalmic centers (all *P* < 0.001). Demographics of the eyes are shown in Table 1.

**Table 1:** Demographics of the subjects in different ophthalmic centers.

	**GZ**	**SY**	**CD**	**WH**	**HK**	***P* ^†^**
Eyes (N)	2340	2255	1480	1511	307	N/A
Eyes (%)	29.65%	28.57%	18.75%	19.14%	3.89%	N/A
Male (%)	46.41%	64.83%	61.49%	50.43%	63.84%	<0.0001
Age (years)^a^	26.94 ± 5.42	23.88 ± 5.14	24.19 ± 5.46	25.39 ± 5.03	23.97 ± 4.78	<0.0001
ACD (mm)^a^	3.27 ± 0.26	3.29 ± 0.26	3.27 ± 0.26	3.21 ± 0.24	3.26 ± 0.25	0.0001
WTW (mm)^a^	11.67 ± 0.38	11.67 ± 0.36	11.67 ± 0.38	11.63 ± 0.38	11.57 ± 0.38	0.0001
SE (D)^a^	−5.17 ± 2.23	−4.81 ± 1.71	−5.27 ± 2.23	−5.28 ± 1.93	−5.65 ± 2.68	0.0001

*N, number of eyes; ACD, Anterior Chamber Depth; WTW, White-to-white; SE, Spherical Error; D, diopter; GZ, Guangzhou Aier Eye Hospital; SY, Shenyang Aier Eye Hospital; CD, Chengdu Aier Eye Hospital; WH, Wuhan Aier Eye Hospital; HK, Hankou Aier Eye Hospital; ^a^Presented as mean ± standard deviation; P-value for comparison among the five groups using Kruskal–Wallis test*.

### Distribution of Corneal Diameter and Anterior Chamber Depth

**Figure 2:**
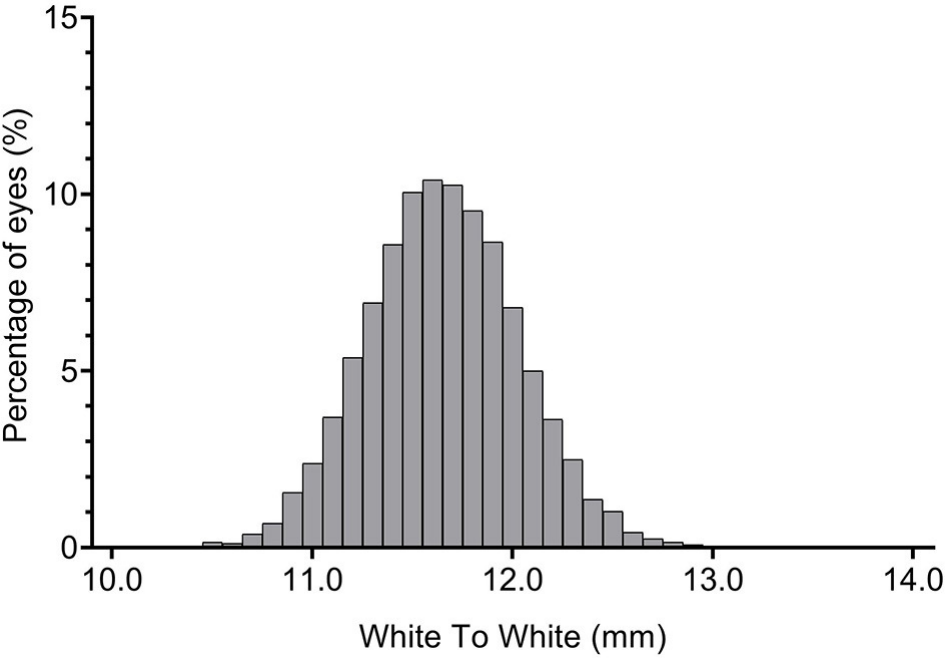
Frequency distribution of white-to-white (WTW) corneal diameter.

**Figure 3:**
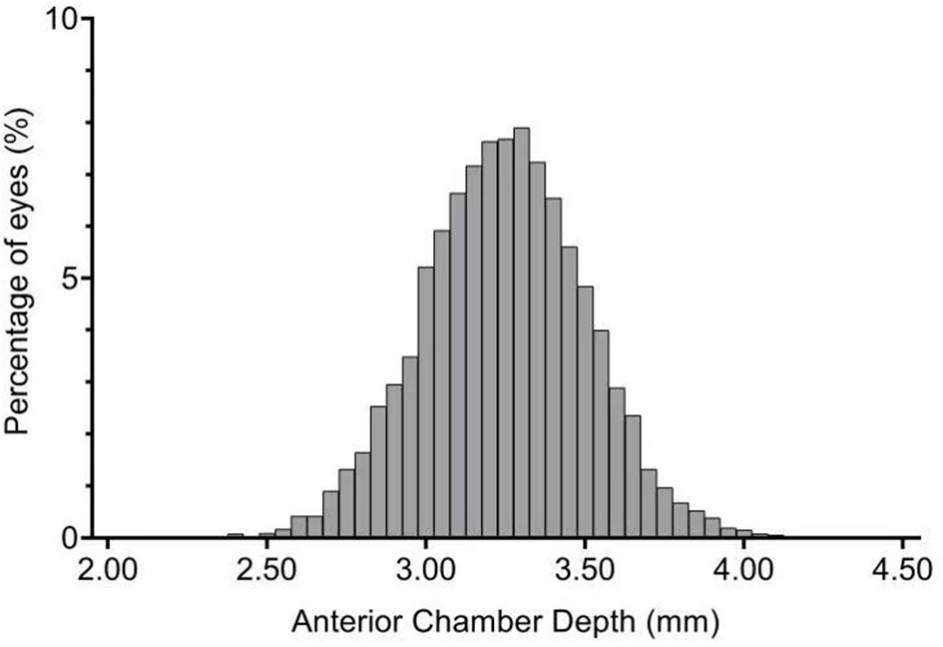
Frequency distribution of anterior chamber depth (ACD).

Distribution of WTW was slightly positively skewed (Figure 2; Skewness = 0.0076, Kurtosis = 0.3944, KS *P* = 0.020); the ACD was normally distributed (Figure 3; Skewness = 0.899, Kurtosis = 0.027, KS *P* = 0.086). The mean WTW was 11.65 ± 0.38 mm (95% CI: 11.64–11.66) and the mean ACD of the study population was 3.25 ± 0.26 mm (95% CI 3.24–3.25). The 95% normal range of WTW in our study was 10.91–12.39 mm, and the 95% normal range of ACD was 2.74–3.75 mm. The smallest WTW was 10.2 mm and the WTW <10.65 mm was seen in 25 eyes (0.32%). The largest WTW was 13.3 mm and the WTW >12.94 mm was seen in 3 eyes (0.04%). Anterior chamber depth in our study population ranged from 2.07 to 4.35 mm. There were 352 eyes (4.46%) with an ACD <2.8 mm and 889 eyes (11.26%) more than 3.55 mm.

Details about the WTW and ACD in different myopia groups are shown in Table 2. Significant differences in WTW and ACD were found among different myopia groups (*P* < 0.001 and *P* = 0.030, respectively). Eyes in the LM group seemed to have slightly lower ACD but significantly larger WTW.

**Table 2:** Anterior chamber depth and white-to-white corneal diameter in different myopia groups of patients.

**Group**	**LM**	**MM**	**HM**	**EHM**	***P***
N (%)	945 (11.97%)	4524 (57.32%)	2272 (28.8%)	152 (1.93%)	/
ACD (mm)^a^	3.22 ± 0.25 (2.73, 3.72)	3.25 ± 0.26 (2.74, 3.75)	3.25 ± 0.25 (2.75, 3.75)	3.25 ± 0.27 (2.73, 3.78)	0.030
WTW (mm)^a^	11.70 ± 0.37 (10.99, 12.42)	11.66 ± 0.38 (10.92, 12.41)	11.61 ± 0.38 (10.87, 12.35)	11.51 ± 0.39 (10.74, 12.27)	<0.001
SE (D)^a^	−2.27 ± 0.49 (−1.32, −3.23)	−4.49 ± 0.83 (−2.87, −6.11)	−7.08 ± 0.89 (−5.34, −8.82)	−12.65 ± 2.97 (−6.83, −18.46)	<0.001

N, number of eyes; ACD, Anterior Chamber Depth; WTW, White-to-white; SE, Spherical Error; D, diopter; LM, low myopia; MM, moderate myopia; HM, high myopia; EHM, extremely high myopia; ^a^Presented as mean ± standard deviation (95% normal range); P-value for comparison among the four groups using Kruskal–Wallis test. *P-value for comparison among the five groups using one way ANOVA test.

Comparison of WTW and ACD between female and male subjects in different myopia groups are shown in Table 3. Significant larger WTW and ACD in males than females were observed in low, moderate and high myopia groups, and significant larger WTW but not ACD in male than female was observed in extremely high myopia group.

**Table 3:** Comparison of anterior chamber depth and white-to-white corneal diameter between different genders among different myopia groups.

**Group**	**LM**		**MM**		**HM**		**EHM**	
	**ACD (mm)^**a**^**	**WTW (mm)^**a**^**	**ACD (mm)^**a**^**	**WTW (mm)^**a**^**	**ACD (mm)^**a**^**	**WTW (mm)^**a**^**	**ACD (mm)^**a**^**	**WTW (mm)^**a**^**
Male	3.26 ± 0.24	11.75 ± 0.36	3.31 ± 0.25	11.75 ± 0.37	3.31 ± 0.25	11.70 ± 0.38	3.29 ± 0.30	11.57 ± 0.40
Female	3.10 ± 0.26	11.57 ± 0.34	3.16 ± 0.25	11.56 ± 0.36	3.19 ± 0.25	11.54 ± 0.36	3.23 ± 0.24	11.46 ± 0.38
*P*	<0.001^†^	<0.001^†^	<0.001^†^	<0.001^†^	<0.001^*^	<0.001^†^	0.082^†^	0.038^*^

ACD, Anterior Chamber Depth; WTW, White to White; LM, low myopia; MM, moderate myopia; HM, high myopia; EHM, extremely high myopia; ^a^Presented as mean ± standard deviation. P-value for ACD and WTW comparison between different genders using two-sample t-test. *P-value for comparison among the five groups using Mann-whitney test.

A significant difference was found among different age groups in terms of ACD and WTW measurements (all *P* <0.001). With increase of age, ACD was shallower and WTW was smaller as showed in Table 4.

**Table 4:** Comparison of anterior chamber depth and white-to-white corneal diameter among different age groups.

**Age (years)**	**ACD (mm)^**a**^**	**WTW (mm)^**a**^**
18–20	3.34 ± 0.23	11.75 ± 0.37
21–23	3.28 ± 0.24	11.68 ± 0.38
24–26	3.25 ± 0.25	11.63 ± 0.36
27–29	3.21 ± 0.26	11.60 ± 0.37
30–32	3.16 ± 0.25	11.55 ± 0.37
33–35	3.13 ± 0.25	11.55 ± 0.35
36–38	3.11 ± 0.27	11.58 ± 0.39
39–40	3.02 ± 0.23	11.55 ± 0.38
*P* ^†^	<0.001	<0.001

*ACD, Anterior Chamber Depth; WTW, White to White. ^a^Presented as mean ± standard deviation. P-value for ACD and WTW comparison between different age groups using Kruskal–Wallis test*.

No significant correlation was found between SE and ACD (*r* = −0.019, *P* = 0.090), or between SE and WTW (*r* = 0.094, *P* < 0.001). A weak but statistically significant negative correlation was found between ACD and age (*r* = −0.286, *P* < 0.001), and between WTW and age (*r* = −0.199, *P* < 0.001).

### Correlation Between WTW and ACD

**Figure 4:**
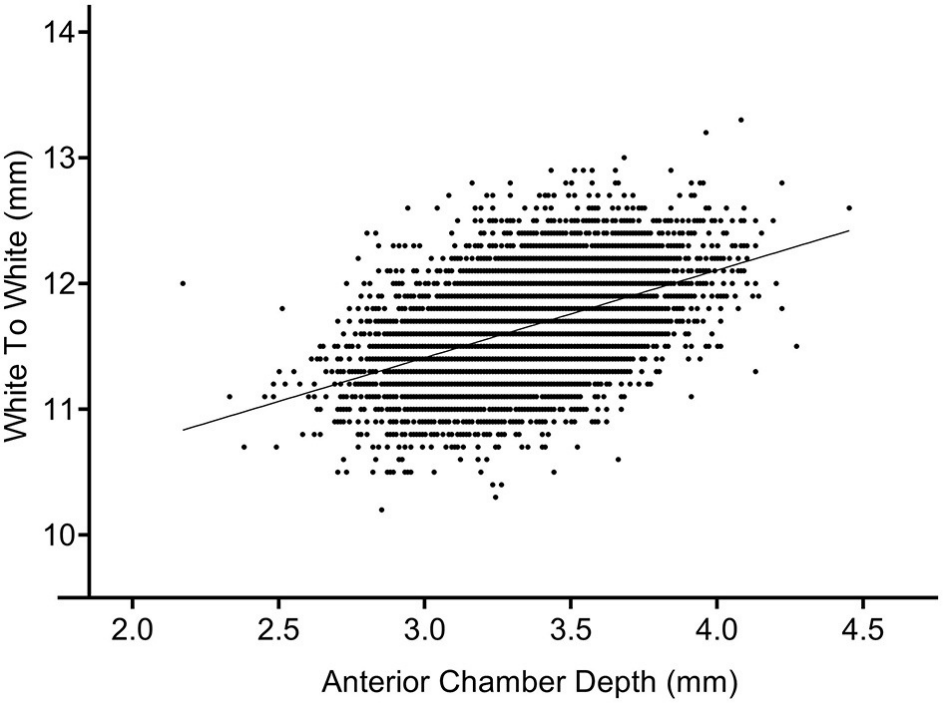
Correlation between anterior chamber depth (ACD) and white-to-white (WTW) corneal diameter.

A significant positive correlation was found between WTW and ACD in our subjects (*r* = 0.460, *P* < 0.001). Patients with shallower anterior chambers might also have smaller WTW (Figure 4).

## Discussions

The distribution patterns of WTW and ACD in myopic patients were investigated in the present multicenter study using pooled data from different ophthalmic centers of mainland China. We found that the average WTW was 11.65 mm and within 10.91–12.39 mm in 95% of eyes. The average ACD was 3.25 mm, and 95.54% of eyes had an ACD of 2.74–3.75 mm. Significant differences in ACD and WTW were found among different myopia groups. The WTW was increased in eyes with deeper ACD.

ICL implantation is a predictable and widely accepted surgery for the correction of myopia (18–20). Although the ICL surgery has been reported to be a relatively safe, effective, alterable, and even reversible surgical approach, a variety of possible complications are associated with the procedure, such as lens opacification, endothelial cell loss, high intraocular pressure, rotation of ICL, and anterior chamber inflammation (7, 21). Postoperative complications also include improper ICL vaulting due to incorrect ICL size selection, which is closely related to preoperative measurement of ACD and WTW (22). ICL sizing for ideal vaulting is challenging. A properly sized ICL can greatly help achieve an optimum vault after the surgery. Patients with a shallower ACD and a smaller WTW tend to have a lower postoperative vault. An over-low vault may increase the risk of lens opacification after surgery. On the contrary, patients with a deeper ACD and a larger WTW are more likely to have a higher postoperative vault. An excessively high vault may cause complications such as pupil ovalization, iritis, pigment dispersion, angle crowding, the liberation of inflammatory mediators, iris chaffing, endothelial cell loss, and angle-closure glaucoma (23). Thus, it is critical to determine the normal ranges of ACD and WTW in myopia eyes so that they can be used as references during ICL sizing.

Currently, the ICL sizes are available for eyes with an ACD of at least 2.80 mm. In our study, 4.46% of eyes had an ACD <2.80 mm. In patients with high myopia and extremely high myopia, 3.59% of eyes had an ACD <2.80 mm. These patients may pose great challenges in ICL size selection. High myopia patients with a shallow ACD (<2.80 mm) are not rare (24). Although it was shown that high myopia patients with shallow ACD achieved satisfying and stable visual outcomes during a follow-up of 15.35 ± 4.90 months after ICL surgery, the long-term safety and stability require further investigation (25).

On the other hand, a shallow ACD is correlated with a higher risk of developing PACG (26). In western countries the majority of glaucoma is the open-angle type. However, the percentage of angle-closure glaucoma in the eastern population is almost 50% (1). The shallower ACD seen in the eastern population due to racial differences might be responsible for the discrepancy in PACG prevalence. Females have shallow ACD as compared to males in the eastern population putting them at higher risks of acute attack of angle-closure and PACG. In the Handan Eye study, 6,830 eligible subjects aged 30 years or older with open-angle were recruited and undergone gonioscopic examinations at baseline and follow-up visits; the results showed that shallow central ACD was a significant risk factor for development of any form of angle closure after 5-year follow up (*OR* = 0.110, *P* = 0.003) (5). In our study, ACD was significantly different among different myopia groups. A previous study showed that a deeper ACD was related to thinner lenses in eyes with longer axial length, which was probably due to geometrical scaling during axial elongation of the eyes (27).

The average WTW in our study was 11.65 ± 0.38 mm, which was similar to Singh et al.'s study conducted in normal Indian subjects (WTW= 11.79 ± 0.67 mm) (1). In our study, the WTW was decreased in patients with the increase of myopia severity (*P* = 0.030), which was in accordance with the study conducted by Zha et al. (28). The decrease of WTW with higher myopia may be due to posterior traction of the limbus caused by elongation of the eyeball. However, further investigations are needed to reveal the exact mechanisms of this finding. Besides ACD, WTW is also of great importance for ICL sizing. Based on Visian ICL product information, patients with WTW <10.65 mm may not be recommended for ICL implantation (24). In our study, 0.32% of myopia eyes had WTW measurement <10.65 mm. For these eyes, development of a new ICL size may be needed.

Both WTW and ACD are important parameters for IOL power selection in cataract surgery. ACD and WTW are indispensable biometric determinants for IOL power calculation formula, such as SRK-T, Holladay 2 and so on (9). Precise preoperative WTW and ACD measurement are significantly associated with accurate IOL positioning after cataract surgery, which is one of the important factors related to satisfactory postoperative visual outcomes (8, 9, 29). In a recent study, larger WTW was observed in younger male patients and eyes with flatter corneas, deeper anterior chambers, thicker lenses, and thinner central corneal thickness (14). The largest WTW was found in eyes with axial length of 24.5 to 26 mm (14). Some of these findings seemed to be different from our results. One of the reasons might be that the populations were different in the two studies. While in Wei et al.'s study the participants were cataractous patients aged 63.7 ± 12.4 years, our study population was young myopic adults with a mean age of 25.14 ± 5.41 years. Different from our study, the WTW and ACD in Wei et al.'s study, might be affected by older age and the cataract (14).

We should be aware that the actual ACD and WTW values may vary according to different measuring instruments such as Orbscan, Pentacam, or IOL Master because of different measuring principles of these instruments. The ACD and WTW measurements of Pentacam may not be interchangeable with Orbscan or IOL Master (30). Although other parameters such as sulcus-to-sulcus (STS) distance on ultrasound biomicroscopy also can be used to calculate the ICL size (31). WTW and ACD were the two major parameters recommended by the manufacturer for choosing ICL size (Visian ICL Product Information: Visian ICL For Myopia. Available at http://www.accessdata.fda.gov/cdrh_docs/pdf3/p030016c.pdf).

What cannot be ignored is that patients with small WTW and narrow ACD may have a higher risk of low ICL vault after surgery (23). In our study, the normal range of ACD was 2.74–3.75 mm, and WTW was 10.91–12.39 mm, indicating a relatively large range of ACD and WTW measurements in myopia eyes. The variety in ACD and WTW may lead to multiple choices in ICL size, and sometimes it is challenging to determine the most appropriate ICL size. Moreover, it remains undetermined about what is a safe ICL vault after surgery. Several studies have reported that 90 μm is the minimum safe vault (32, 33). Gonvers et al. recommended a central vault of 150 μm, to protect the lens from contact with the ICL (34). Choi et al. reported an ideal ICL vault to be 250–750 μm (35). The maximum safe vault may be associated with preoperative ACD. A high preoperative ACD is likely to render a high vault after ICL implantation, and may have better tolerance for high ICL vault postoperatively. However, an ideal ACD which could predictive a safe range of ICL vault is still under investigation (36). Importantly, it is necessary to take into accounts the effects of accommodation on vaulting and the age-related reduction of the central vault when selecting the ideal ICL size.

Our study has some limitations. Firstly, we only included myopic patients in the study. Distribution of ACD and WTW in emmetropic and hyperopic subjects needs to be determined in future investigations. Secondly, our results can only be applied to a relatively young age group (18–40 years). In older patients the distribution pattern of ACD is significantly different, although the difference in WTW with aging may be less significant. Thirdly, we did not collect axial length (AL) data, and the relationship between AL and ACD/WTW could not be assessed in our study. Since the AL is better indicator of growth of the eye in myopia compared to the SE, it is important to evaluate the ACD and WTW distribution in eyes with different AL. Lastly, the study subjects in the five ophthalmic centers were inhomogeneous in terms of age and gender which might have some impact on the results, but we believe that the current results maybe more representative of the WTW and ACD in a real world scenario, instead of a specific “uniform” population.

In conclusion, we demonstrated the distribution patterns of ACD and WTW in Chinese myopic patients using multicenter data. The 95% normal range for ACD was 2.74–3.75 mm and 10.91–12.39 mm for WTW. ACD and WTW were significantly different among different myopia, gender and age groups. WTW was positively correlated with ACD.

## Data Availability Statement

The raw data supporting the conclusions of this article will be made available by the authors, without undue reservation.

## Ethics Statement

The study was approved by the Institutional Review Board (IRB) of every hospital (GZ, SY, CD, WH, and HK) and conducted in agreement with the Declaration of Helsinki. Written informed consent for participation was not required for this study in accordance with the national legislation and the institutional requirements.

## Author Contributions

GX and YH designed the study and wrote the manuscript. GX, GW, ZD, SZ, and YG organized and analyzed the data and commented on the manuscript. HY and YH supervised the study and edited the manuscript. All authors contributed to the article and approved the submitted version.

## Funding

This work was supported by Grant A2021378 from the Medical Scientific Research Foundation of Guangdong Province, China (YH), Grant 2018SK50106 from the Technology Innovation Guidance Program of Hunan Province (YH), Grant AM1909D2 and AR1909D2 from the Science Research Foundation of Aier Eye Hospital Group (YH).

## Conflict of Interest

The authors declare that the research was conducted in the absence of any commercial or financial relationships that could be construed as a potential conflict of interest.

## Publisher's Note

All claims expressed in this article are solely those of the authors and do not necessarily represent those of their affiliated organizations, or those of the publisher, the editors and the reviewers. Any product that may be evaluated in this article, or claim that may be made by its manufacturer, is not guaranteed or endorsed by the publisher.
